# Methods for estimating tuberculosis incidence and mortality by age and sex

**DOI:** 10.1093/ije/dyaa257

**Published:** 2021-02-24

**Authors:** Peter J Dodd, Charalambos Sismanidis, Philippe Glaziou

**Affiliations:** 1 School of Health and Related Research, University of Sheffield, Sheffield, UK; 2 Global TB Programme, World Health Organization, Geneva, Switzerland

## Abstract

**Aims:**

To develop methods to disaggregate World Health Orgagnization estimates of tuberculosis (TB) incidence and mortality for each country by sex and age.

**Methods:**

For countries where incidence estimates derived from a factor adjustment of notifications and case detection ratio over 0.85, or with <1000 reported TB cases, we disaggregated incidence proportional to notifications. For each other country, a prior was constructed using a hierarchical model of age-stratified prevalence survey data, meta-analysis of sex ratios, and mathematical modelling for children under 15 years. Samples from this prior were used to disaggregate incidence and accepted if incidence exceeded notifications in each age/sex category. Results were inspected and, if implausible, incidence was disaggregated proportional to notifications. Mortality was disaggregated proportional to patterns in vital registration (VR) data in countries with VR data. Where VR data were lacking, a case-fatality ratio (CFR) approach was applied to estimated incidence, with separate CFRs by HIV/ART status, child/adult age groups, and anti-TB treatment status. Uncertainty in all disaggregated country estimates was constructed to be consistent with corresponding overall uncertainty.

**Results:**

We generated disaggregated results for 216 countries. For 125 countries, incidence disaggregation was based on notifications. Of the rest, accepted samples from the prior were considered implausible in 4 countries. For 72 countries, mortality disaggregation was based on VR data; the rest were based on the CFR approach.

**Conclusions:**

While multi-stage, this approach is comparatively simple in overall logic. Disaggregated estimates have relatively larger uncertainty and should be used with caution.

## Introduction

Despite modest declines in global incidence, tuberculosis (TB) remains the world’s leading cause of death from a single pathogen.^1^ The tuberculosis monitoring and evaluation unit of the Global TB programme at the World Health Organization (WHO) undertakes a yearly analysis of data supplied by its member states to generate estimates of TB burden in each state, regionally and globally. These estimates are released after a country consultation process as part of the WHO annual Global Tuberculosis Report, which provides analysis for the previous year.^1^ Estimates of TB disease burden are used to monitor progress towards targets as part of the End TB strategy, as well as to provide evidence on the scale and distribution of TB to inform intervention strategies and investment cases.

TB incidence estimation is necessary, as sample size and expense preclude general population surveys of TB incidence. In some settings with strong surveillance systems, the majority of TB cases are diagnosed and reported and notification data are a close approximation to underlying incidence. In such settings, a common ‘standard factor’ adjustment is applied to scale up notifications to incidence (unless an inventory study can inform a local factor). However, in many settings, under-diagnosis and under-reporting each contribute to a substantial gap between underlying incidence and notifications, amounting to approximately^2^ million cases globally in 2018.^1^ Data from TB prevalence surveys and inventory studies of under-reporting of detected TB cases, as well as expert opinion based on epidemiological assessments, inform TB incidence estimates in these settings. Similarly civil and vital registration systems provide direct data on the number of TB deaths, but these are not available for many countries. For these countries, indirect estimates of TB mortality are based on estimates of the case fatality ratio (CFR) in different groups. The methods used for these estimates are described in the Global Tuberculosis Report and its technical appendix.^1^^,^^2^

There is an increasing demand for more finely-grained estimates of TB burden, stratified by age group and sex, to identify under-served groups and tailor public health strategy to local epidemiology. For example, data from surveillance and prevalence surveys suggest that TB burden is higher in men in most settings.^3^ In some settings a high burden of TB is born by older age groups,^4^ whereas in many high-burden settings, children under 15 are thought to be relatively under-diagnosed.^5^ Since 2017, Global Tuberculosis Reports have generated estimates of TB incidence and mortality disaggregated by age and sex for the most recent year.^1^^,^^6^^,^^7^ This builds on previous work reporting estimates for children separately from adults, first included in the 2012 report,^8^ and for which specific adjustment and modelling approaches handle the relatively larger gap between incidence and notifications for children under 15 years of age, thought to exist in many settings.^5^^,^^9^^,^^10^

This article details the current approach used to generate age- and sex-disaggregated estimates of TB incidence and mortality for the Global Tuberculosis Report.^1^

## Methods

We developed a methodology to disaggregate estimates of tuberculosis incidence and mortality by sex and age categories: 0–4, 5–14, 15–24, 25–34, 35–44, 45–54, 55–64 and 65+ years, and applied it to the most recent year’s data (here 2018). To ensure consistency with envelope uncertainty, age- and sex-stratified variances split country-level variances in proportion to each stratum point estimate.

### Incidence disaggregation

To disaggregate tuberculosis incidence, we followed three steps, guided by estimated case detection ratio (CDR, the ratio of notifications to estimated incidence).

These countries were those with either strongly performing surveillance systems that could reliably inform on patterns by age and sex, or those where the epidemic is small enough for true patterns to be driven by stochastic and import effects, making modelled predictions less appropriate. We used the most recent age-disaggregated notification data if the previous year’s data were unavailable as follows.

1) For countries where either:
 (i) standard factor adjustment was used to estimate tuberculosis incidence and CDR >0.85;
 (ii) a capture-recapture study was used to estimate tuberculosis incidence and CDR >0.85; or
 (iii) fewer than 1000 tuberculosis cases were reported in total;
 we disaggregated total tuberculosis incidence by age and sex in proportion to the notifications.

2) We drew 1 million samples from a country prior for the proportion of tuberculosis incidence in each age and sex category (see next section for details). Samples were accepted if they yielded incidence higher than notifications in every category or, if no samples met this criterion, the 1% of samples with the smallest sum of squared undershoots was used (undershoots being those differences between incidence and notifications which are negative). We then disaggregated according to the mean proportions accepted samples.
3) For countries where the results from 2) above were judged implausible based on inconsistency with notification data (e.g. undershoots) or feedback from country experts, we disaggregated in proportion to notifications.

To visually assess outputs, we checked plots for each country of incidence and notifications by age and sex.

### Construction of priors

The prior probability distributions for the proportion of tuberculosis in each age and sex category in each country were constructed based on tuberculosis prevalence survey data, systematic review and modelling of paediatric incidence.

The prior for the proportion of adult incidence in each age category was based on a Bayesian hierarchical model of prevalence survey data stratified by WHO region. In total, we used 24 nationally representative TB prevalence surveys since 2010 which reported bacteriologically confirmed TB prevalence (with confidence intervals) for age categories 15–24, 25–34, 35–44, 45–54, 55–64 and 65+ years. For each region, the logarithmic relative risk of tuberculosis prevalence in each age category (with appropriate sample uncertainty) was modelled as a multivariate normal with a regional normal inverse-Wishart prior. This enabled meta-analytical predictions for countries in a region without surveys, and more precise and local estimates for countries with survey data.

More formally, we took the observed vector *z_i_* of logarithmic relative risks by age in country *i* (where i=1,…,n), to be distributed $z_{i}∼\mathrm{MVN}(y_{i},E_{i})$, where *E_i_* is a specified diagonal variance matrix with elements determined by the confidence intervals associated with each element of *z_i_*, and where the mean *y_i_* is the underlying vector of true logarithmic relative risks by age in country *i*. We modelled *y_i_* as multivariate normal:

$$y_{i}∼\mathrm{MVN}(\mu ,\Sigma ),$$

with a shared normal inverse-Wishart prior across each region, i.e.

$$\mu ,\Sigma ∼\mathrm{NIW}(\mu_{0},\lambda ,\Psi ,\nu ).$$

The normal inverse-Wishart is conjugate to the multivariate normal distribution so that the conditional distribution for μ,Σ|Y (where $Y={\left\{y_{i}\right\}}_{i=1}^{n}$) is also normal inverse-Wishart, with new parameters $\mu_{0}^{\prime },\lambda \prime ,\Psi \prime ,\nu \prime$ determined by a standard update rule.^11^ We sampled from the overall model using a Gibbs sampling scheme that alternated this step updating the distribution μ,Σ|Y with a step sampling Y|μ,Σ,Z (where $Z={\left\{z_{i}\right\}}_{i=1}^{n}$). This second update can also be performed easily since:

$$y_{i}|\mu ,\Sigma ,Z∼\mathrm{MVN}\left((z_{i}E_{i}^{-1}+\mu \Sigma^{-1})S_{i},S_{i}\right),$$

where $S_{i}^{-1}=(E_{i}^{-1}+\Sigma^{-1})$.

The split of tuberculosis incidence between male and female adults (MF ratio) was based on Horton *et al*.’s systematic review and meta-analysis of tuberculosis sex-ratios.^3^ The age-controlled relative risk of TB by sex in a country was numerically solved to match the meta-analysis sex ratio for the corresponding WHO region (or the country-specific MF ratio if informed by prevalence survey data), once weighted by adult demographic patterns.

In more detail: let *σ_s_* be the underlying relative TB risk for sex s∈{f,m} for females and males, respectively (taking females as the reference category), and *ρ_a_* be the underlying relative TB risk for age category *a* (relative to 15–24 year olds), and model relative risk age *a* and sex *s* multiplicatively as $\sigma_{s}\rho_{a}$. With $N_{a,s}$ the population in a country for age category *a* and sex *s*, the observed marginal relative risks *RR_a_* for age *a* and *MF* (the male: female prevalence ratio) will be given by:

$$\begin{array}{c} RR_{a}=\frac{\sum_{s}N_{a,s}\sigma_{s}\rho_{a}/N_{a}}{\sum_{s}N_{15-24,s}\sigma_{s}\rho_{15-24}/N_{15-24}}\\ MF=\frac{\sum_{a}N_{a,m}\sigma_{m}\rho_{a}/N_{m}}{\sum_{a}N_{a,f}\sigma_{f}\rho_{a}/N_{f}} \end{array}$$

where *N_a_* and *N_s_* are the total number of people in this country for age category *a* and sex *s*, respectively. In general, *MF* will be somewhat different from *σ_m_* and *RR_a_* somewhat different from *ρ_a_*. We numerically solved these equations for *σ_m_* and *ρ_a_* given *MF* and *RR_a_*, and evaluated the joint proportion of adult TB cases in age category *a* and sex *s* as:

$$P_{a,s}=\frac{\rho_{a}\sigma_{s}N_{a,s}}{\sum_{a,s}\rho_{a}\sigma_{s}N_{a,s}}$$

The prior fraction of all tuberculosis incidence among children under 15, and the fraction among children under 5, were sampled from a previous approach to paediatric tuberculosis estimation based on modelling transmission from adults and progression to disease,^5^ using updated input data. The sex splits in 0–4 and 5–14 age groups were based on a WHO region-stratified random effects meta-analysis of the ratios in the most recent notification data.

### Mortality disaggregation

As with incidence, disaggregating mortality for a country depended on the approach used to estimate total tuberculosis mortality in that country. For countries whose VR data were rated high and medium quality for the Sustainable Development Goal,^12^ the proportion of deaths recorded in each age and sex category was used to disaggregate estimated deaths. For International Classification of Diseases (ICD)-10, codes A15-A19 and B90 were used; for ICD-9, codes 010–018 and 137 were used.

For countries where estimates of total tuberculosis mortality were based on a case-fatality ratio (CFR) approach, the disaggregation of estimated mortality used a CFR-based estimate of the proportion of deaths in each age- and sex-category. For countries with CFR-based estimates of mortality, literature estimates of CFR stratified by human immunodeficiency virus (HIV), antiretroviral treatment (ART) and TB treatment status were used to estimate separately tuberculosis mortality among HIV-uninfected and HIV-infected individuals.

For children aged <15 years, we used a previously published CFR approach to estimate tuberculosis mortality in children,^13^ stratifying CFR by age (0–4 and 5–14 years) and TB treatment status (estimated from estimated incidence and notifications), based on systematic review,^14^ in addition to HIV, ART status. This enabled estimation of the fraction of all tuberculosis deaths occurring in children aged <15 years, and by age category, sex and HIV status. For adults (aged 15+ years), separate CFRs were used for HIV-infected and HIV-uninfected individuals, which were assumed to be constant across all adult age groups and both sexes.

To visually assess outputs, we checked plots for each country of the fraction of tuberculosis mortality in each age and sex group.

## Results

In this article, we report results from intermediate steps in the analysis and relevant to illustrating the performance of the methodology. The main results are reported in the WHO Global Tuberculosis Report 2019 and associated data.^1^

### Hierarchical model of prevalence survey data

**Figure 1:**
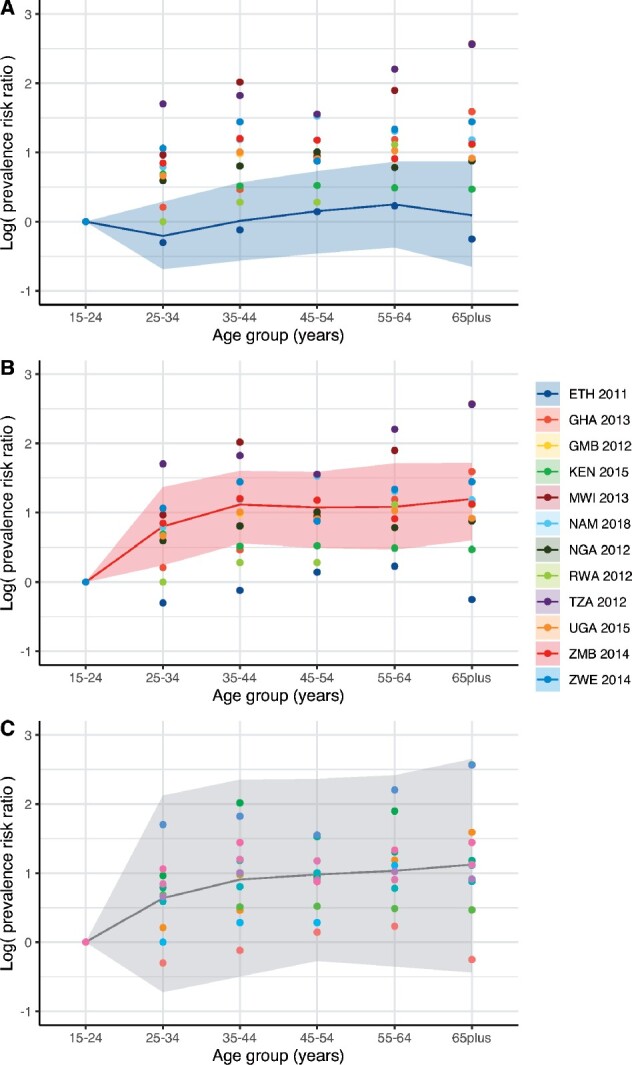
Hierarchical model of TB prevalence survey data in the WHO Africa region. Prediction (posterior median) and uncertainty bands (95% credible interval) from fitted hierarchical model for countries with surveys: A) Ethiopia, B) Zambia; and C) for countries in this region without data

The regionally stratified hierarchical model of age patterns used in constructing our incidence prior generated predictions that followed patterns in available country data, slightly pulling predictions towards the regional mean (see Figure 1a and b for results for the WHO Africa region). The predictions for counties in a region without survey data reflected a weighted mean, and the larger uncertainty intervals captured most survey data (see Figure 1c).

### Notification sex ratios in children under 15 years

A random effects meta-analysis of the fraction of notifications by sex for those those aged under 5 years used in constructing our incidence prior found 54.5% [95% confidence interval (CI) 53.7%, 55.4%] and [95% prediction interval (PI) 48.6%, 60.3%] reported cases were male. For cases aged 5–14 years, 49.5% (95% CI 48.7%, 50.4%) and (95% PI 42.6%, 56.5%) cases were male. A random-effects meta-regression using WHO region as an explanatory variable found these fractions were consistent across regions, see Table 1.

**Table 1:** Fraction of TB notifications among males for children under 15 years. Central estimates and 95% prediction intervals from random effects meta-regression with WHO region as covariate, and without covariates for the global result

WHO region	Fraction male: age 0-4 years (95% prediction interval)	Fraction male: age 5-14 years (95% prediction interval)
AFR	0.534 (0.488, 0.591)	0.497 (0.430, 0.565)
AMR	0.543 (0.488, 0.596)	0.485 (0.415, 0.555)
EMR	0.550 (0.495, 0.604)	0.475 (0.406, 0.546)
EUR	0.522 (0.466, 0.577)	0.507 (0.437, 0.577)
SEA	0.569 (0.515, 0.621)	0.508 (0.437, 0.578)
WPR	0.566 (0.488, 0.591)	0.499 (0.429, 0.570)
Global	0.545 (0.486, 0.603)	0.495 (0.426, 0.565)

The WHO regions are AFR = Africa, AMR = The Americas, EMR = Eastern Mediterranean, EUR = Europe, SEA = South-East Asia, WPR = Western Pacific

### Incidence results

In total, we disaggregated tuberculosis incidence for 216 countries. For 125 countries (13% of global incidence), this used notification data (Method 1). Of these: 28 countries (13.0% of global incidence) had incidence estimates based on standard factor adjustment and CDR >0.85; two countries (4.1% of incidence) had incidence estimates based on inventory studies and had CDR >0.85; and 95 countries (0.2% of global incidence) had fewer than 1000 tuberculosis reported cases in total. (We classed countries as having fewer than 1000 reported tuberculosis cases if they also met another criterion for Method 1.)

For 89 countries (53.5% of global incidence), this was based on the prior/rejection approach (Method 2). For three countries (0.2% of global incidence)—South Sudan, Syrian Arab Republic, Togo—disaggregated estimates were based on samples where incidence undershot notifications in at least one age and sex category.

Method 3: for four countries—Bangladesh, India, Myanmar, Democratic People’s Republic of Korea—(33.5% of global incidence), we reverted to a standard factor adjustment, based on the implausibility of outputs from Method 2. The prior struggled to reflect observed sex ratio (India, Bangladesh) and/or predicted insufficient incidence among younger adults (Bangladesh, India, Myanmar, Democratic People’s Republic of Korea). See Appendix (available as Supplementary data at *IJE* online) for plots comparing incidence disaggregation results by Methods 2 and 3 for these countries.

**Figure 2:**
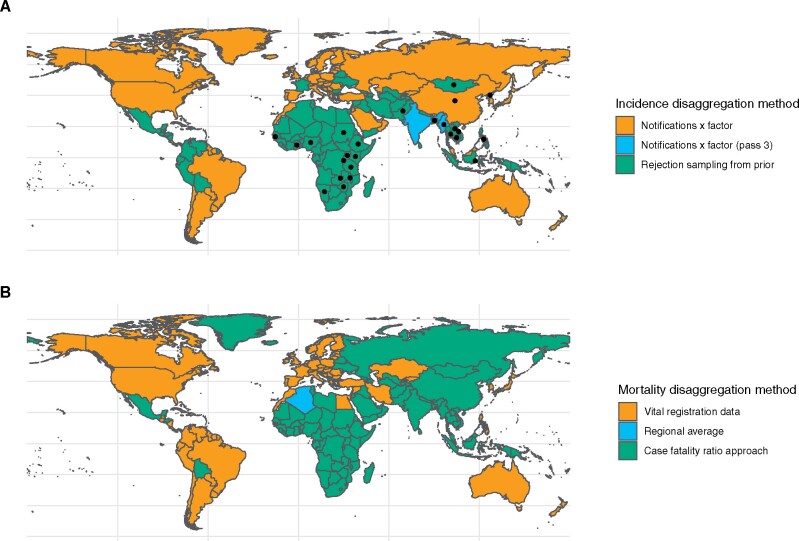
Map of methods used for disaggregation of TB incidence (A) and mortality (B). Colours represent method (described in text). Dots mark countries with a recent national TB prevalence survey whose data has been used in the hierarchical model prior

**Figure 3:**
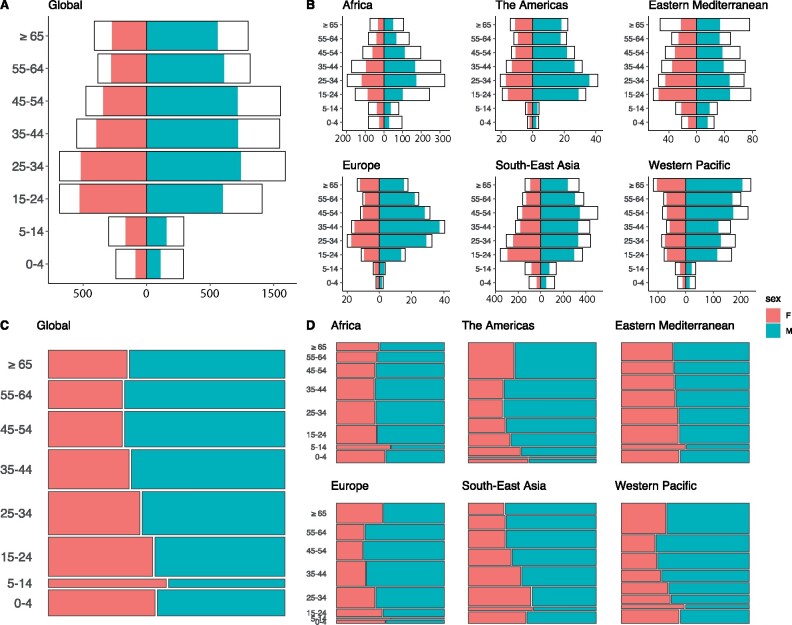
Global and regional aggregate patterns of TB incidence (panels A and B; x-axes in thousands per year, y-axes show age groups in years) and TB mortality (panels C and D; y-axes show the proportion of deaths in each age group, x-axes show the proportion of deaths in each sex within each age group). Red (left) represents females; green (right) represents men

Figure 2a shows the method used for incidence disaggregation in each country. Figure 3a and b shows incidence estimates and notifications by age and sex group, aggregated at global and WHO region level, respectively.

### Mortality results

In total, we disaggregated tuberculosis mortality for 216 countries. For 72 countries (4.3% of global HIV-negative TB mortality), mortality disaggregation was based on VR data. For 117 countries (95.4% of global HIV-negative TB mortality), mortality disaggregation was based on the CFR approach. For 27 countries, (0.3% of global HIV-negative TB mortality), mortality disaggregation was based on regional average patterns.

Figure 2b shows the method used for mortality disaggregation in each country. Figure 3c and d shows estimated mortality fractions in each age and sex group, aggregated at global and WHO region level, respectively.

## Discussion

This article describes a methodology for disaggregating national estimates of TB incidence and mortality by age and sex. This has evolved over the past 3 years,^1^^,^^6^^,^^7^ and the process presented here corresponds to that used for burden estimates in the 2019 Global Tuberculosis Report.^1^ Methods are likely to evolve in the future, responding to new evidence, data and identified shortcomings.

The methods presented here do not fall within a single formal statistical framework, but do adhere to a set of principles. The first is to make use of robust, local surveillance data wherever possible. WHO member states provide input data, and expect it to be reflected in country estimates. Therefore, where country VR data are available, mortality disaggregation has been based on these. Where the estimated gap between incidence and notifications is comparatively small, the disaggregation of incidence has been based on this. Where this gap is larger, however, notifications may not be a reliable guide to relative burden in each age group and identifying comparatively under-served groups necessitates alternative approaches.

The second principle is to seek to maintain simplicity and transparency. The variety in epidemiological patterns, surveillance biases and data completeness across 216 countries make this challenging. We prefer explicit use of *ad hoc* approaches (e.g, the third step of the incidence disaggregation approach) to rigid adherence to a formalism whose outputs lack credibility and buy-in among those using them or providing the data on which they are based.

Last, we require estimates and their associated uncertainty to be robust and consistent. Whereas estimates should reflect true trends and inevitably shift with methodological innovation and new evidence, robustness means that estimates should not be unduly sensitive and fluctuate over time or between rounds without clear justification. Consistency requires that aggregating estimates and uncertainty across countries and any set of categories generates results that match estimates at the aggregate level. Building up total estimates from each subcategory is in principle possible, but category-specific supporting data are more often lacking. We enforced consistency under a disaggregation approach—modelling estimates in each category and their uncertainty as a proportion of each country’s total. This is similar to disaggregation of mortality estimates across causes of death, where the total envelope is known more accurately than individual causes.

Our approach considers each country and TB incidence and TB mortality separately, and does not guarantee consistency of relationships between quantities or locations. This contrasts with the approach used by the Institute of Health Metrics and Evaluation (IHME), who estimate TB incidence and mortality by age, sex and country within a single meta-analytical framework alongside health burdens due to other causes.^15^ Their approach may allow more efficient inference from the available data by borrowing strength across measures, causes and locations, but makes it harder to explain specific results where data are sparse. IHME global estimates are similar in pattern to WHO estimates for incidence, but have higher proportions of deaths in older age groups (see Appendix), presumably reflecting stronger weighting for patterns in VR data. Estimates of TB burden from the Global TB Department of WHO feed into the Global Health Observatory data produced by the WHO and associated World Health Statistics reports.^12^^,^^16^ These include a range of causes of morbidity and mortality, and may require adjustments to disease-specific causes to reconcile totals with envelopes.

This approach has many limitations. These include the principle that incidence should exceed notifications; in some countries and age categories over-diagnosis is possible, particularly when the proportion of bacteriologically confirmed cases is low and there is over-reliance on clinical diagnosis. The childhood notification data suffer from serious limitations, including inconsistencies in applied diagnostic criteria: some notified cases may have been wrongly diagnosed as childhood TB cases, and under-reporting of detected cases and under-diagnosis remain common where access to quality health services is lacking. The prior for adult age patterns is based on prevalence data; differential duration of disease may mean these are biased with respect to incidence patterns. Furthermore, our prior did not allow interactions between sex and age patterns, and adult CFRs for untreated TB did not vary by age or sex. CFRs for untreated TB may increase with age, which would mean our assumption under-estimates TB deaths in older age groups. Last, we have not considered within-country heterogeneity; subnational correlations in relevant quantities could bias estimates in either direction.

Anticipated updates to this process include producing disaggregated estimates for all years and additional disaggregation for age groups 5–14 and 15–24. These age groups reflect traditional groupings for TB notification data, but 5-year splits would allow easier comparison with other estimates and are relevant to an increased emphasis on adolescent health.^17–19^ Evidence on case fatality rates by age and sex would be helpful for countries using a CFR approach to mortality disaggregation. Data from six new national prevalence surveys during 2019–29 are expected, including India, and will be incorporated into the hierarchical model for age pattern priors.

For estimates such as these, the smaller the population, the larger the relative uncertainty, implying that age-, country- and sex-specific estimates should be interpreted with caution and not used for target setting. However, these estimates may serve as a guide in identifying population groups with higher burden or poorer case detection which warrant further attention for investigation or intervention. Ultimately, the best way to improve estimates of disease burden is through strengthened surveillance and reporting systems, including development of civil and vital registration systems.

## Supplementary data

Supplementary data are available at *IJE* online.

## Funding

This work was funded by an MRC fellowship to P.J.D. (MR/P022081/1); this UK-funded award is part of the EDCTP2 programme supported by the European Union.

## Conflict of interest

None.

## Supplementary Material

dyaa257_Supplementary_Data
