# Supplementary material for: Methods for estimating tuberculosis incidence and mortality by age and sex
Source: Int J Epidemiol. 2021 Feb 24;50(2):570–7. doi: 10.1093/ije/dyaa257 (PMC8128472; doi:10.1093/ije/dyaa257)
Supplement: dyaa257_Supplementary_Data [file dyaa257_supplementary_data.pdf]

Methods for estimating tuberculosis incidence and mortality by age and sex: supplementary appendix

Peter J. Dodd[1*], Charalambos Sismanidis[2], Philippe Glaziou[2]

1. School of Health and Related Research, University of Sheffield, Sheffield, UK
2. Global TB Programme, World Health Organization, Geneva, Switzerland

* corresponding author: p.j.dodd@sheffield.ac.uk, 30 Regent Street, Sheffield, S1 4DA, UK

Figure S 1: MCMC chain plot for the top-level MVN mean parameters (WHO Africa region)........... 2
Figure S 2: MCMC chain plot a specific country's MVN mean parameters (in WHO Africa region). 2
Figure S 3: MCMC chain plot for predicted log prevalence ratios (WHO Africa region) 3
Figure S 4: Output corresponding to Figure 1 for the WHO SEA region 4
Figure S 5: Output corresponding to Figure 1 for the WHO WPR region 5
Figure S 6: Output corresponding to Figure 1 for the WHO EMR region 6
Figure S 7: The countries where Method 2 did not result in acceptable estimates............................... 7
Figure S 8: Comparison of IHME (dots, estimates for 2017) and WHO (bars, estimates for 2018) patterns of TB incidence (panel A) and deaths (panel B) by age and sex. 8

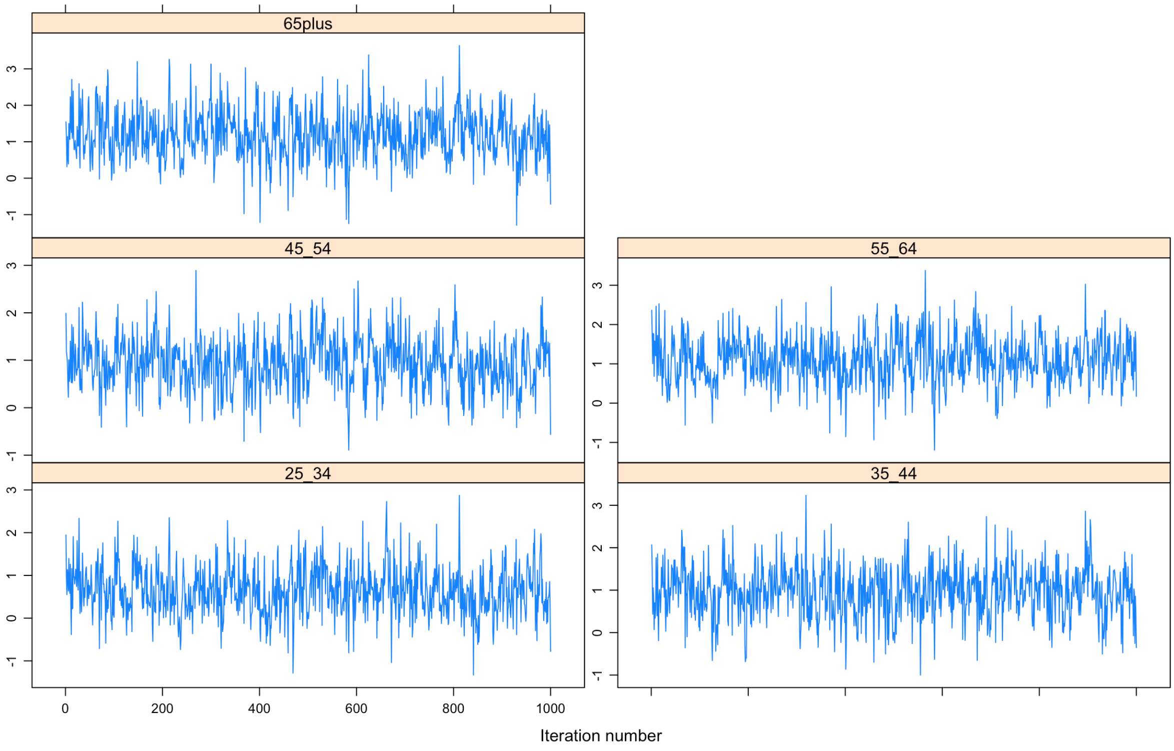

Figure S 1: *MCMC chain plot for the top-level MVN mean parameters (WHO Africa region)*

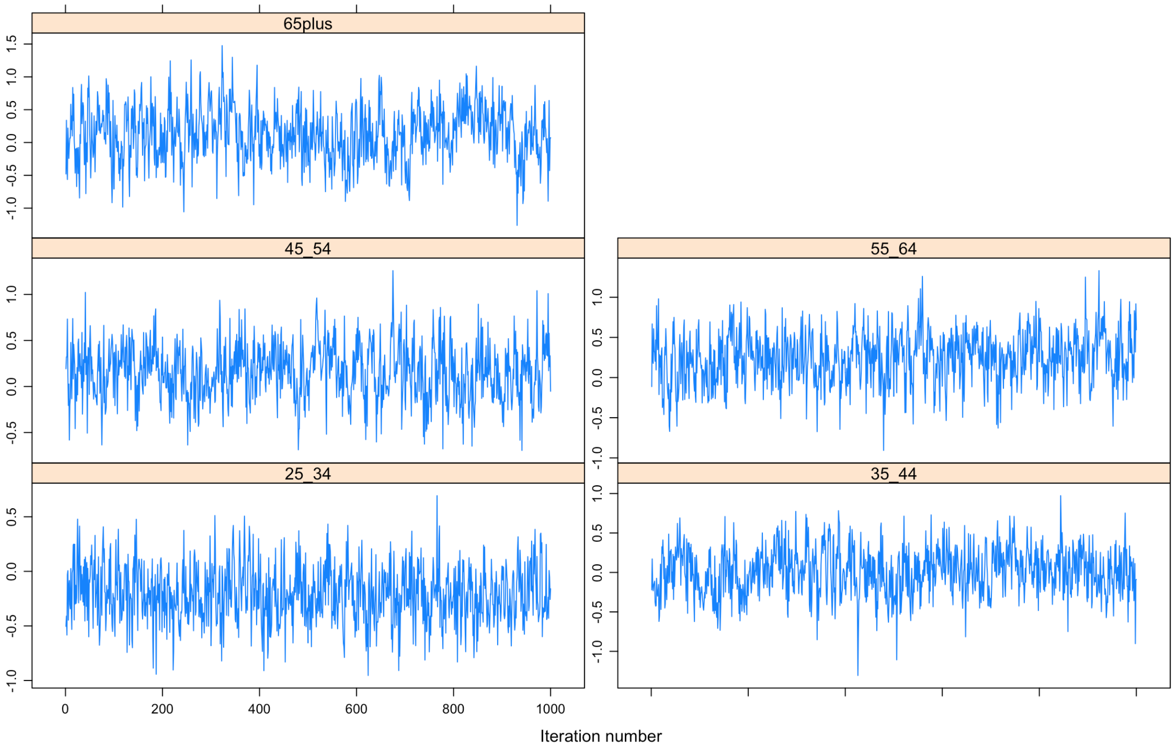

Figure S 2: *MCMC chain plot a specific country's MVN mean parameters (in WHO Africa region)*

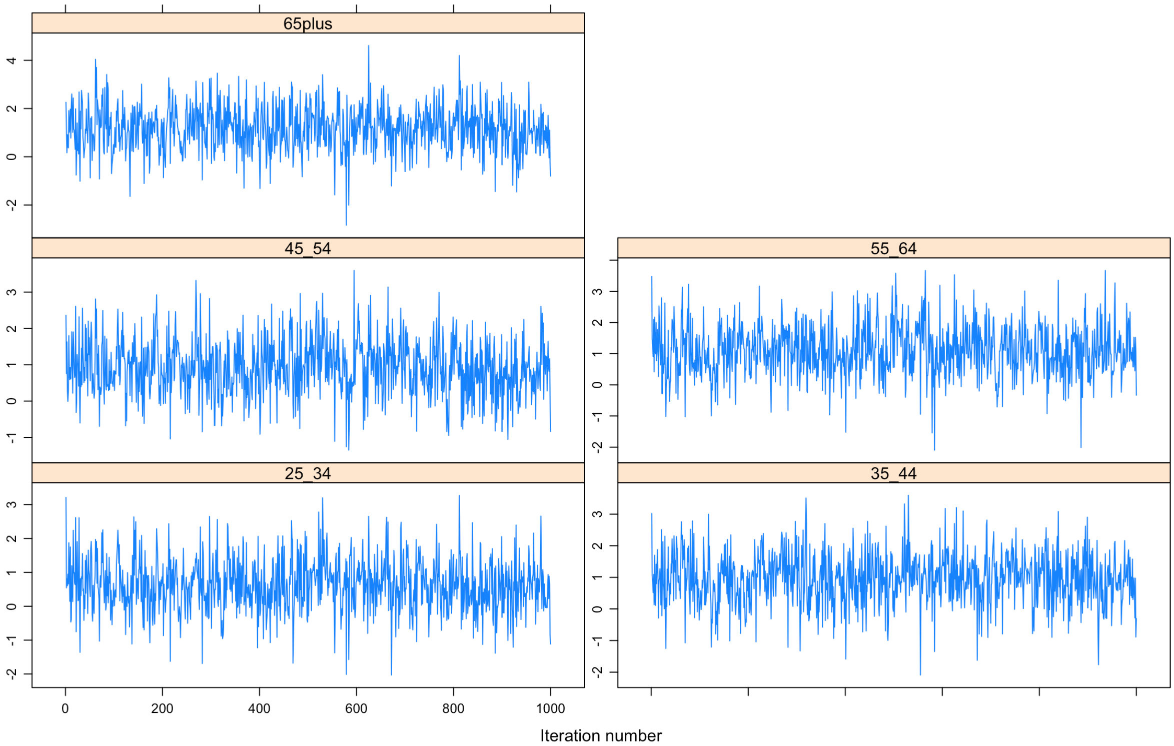

Figure S 3: *MCMC chain plot for predicted log prevalence ratios (WHO Africa region)*

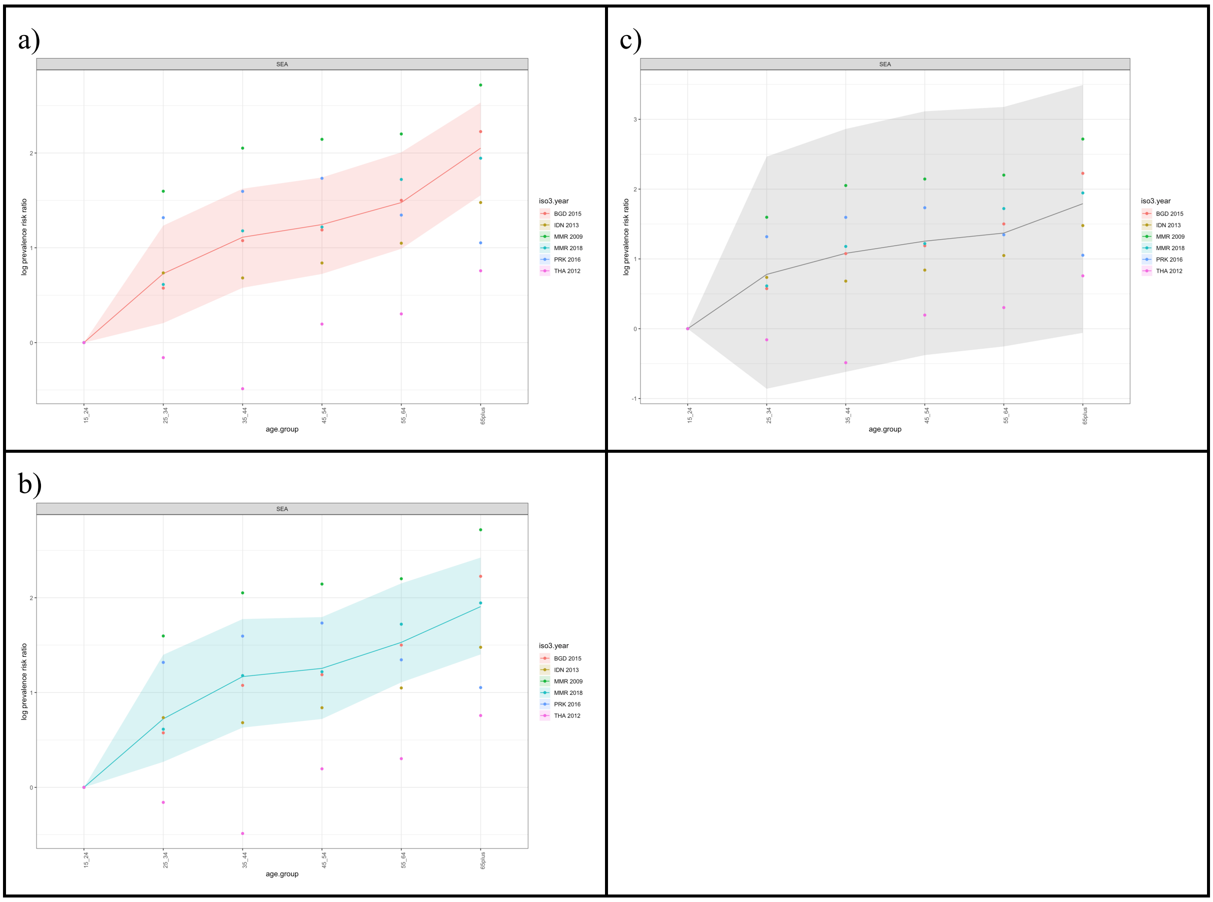

Figure S 4: *Output corresponding to Figure 1 for the WHO SEA region*

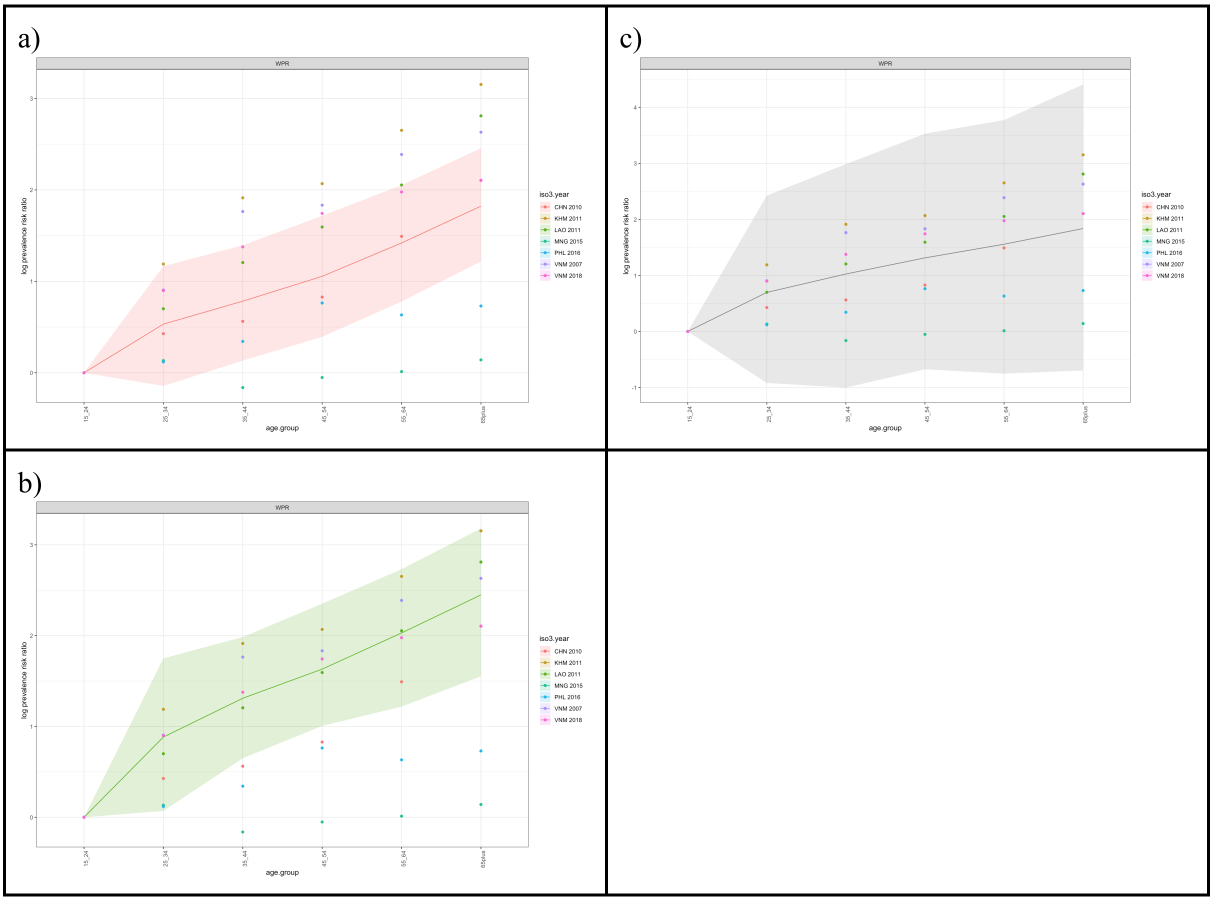

Figure S 5: *Output corresponding to Figure 1 for the WHO WPR region*

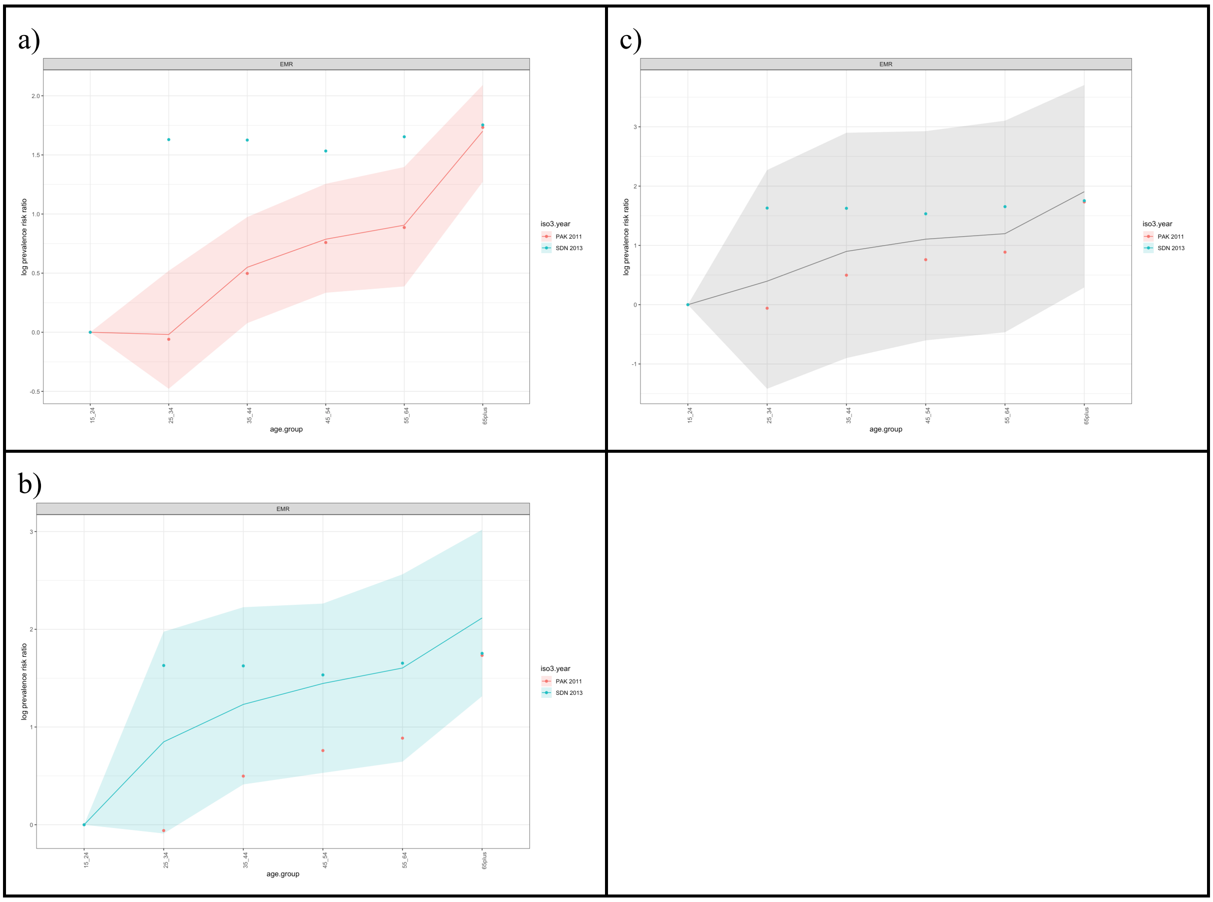

Figure S 6: *Output corresponding to Figure 1 for the WHO EMR region*

Country	Method 2	Method 3
Bangladesh	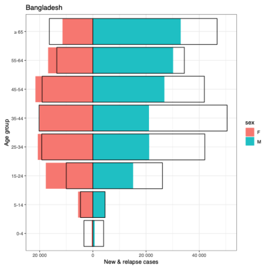	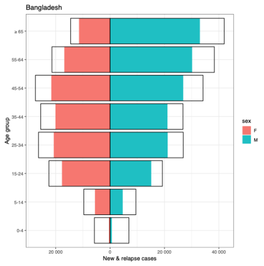
China	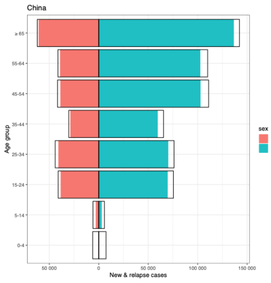	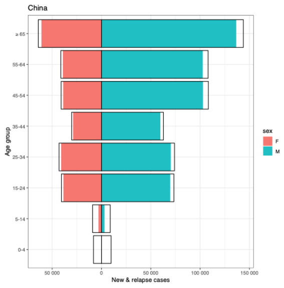
India	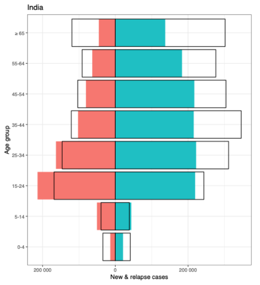	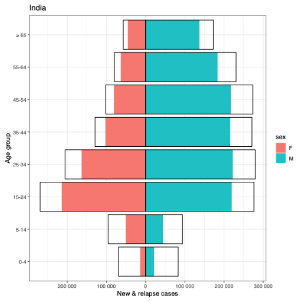
Myanmar	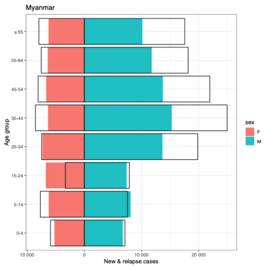	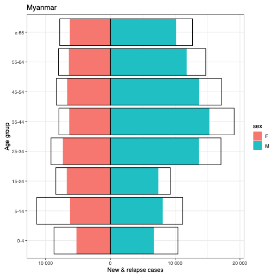
Democratic People's Republic of Korea	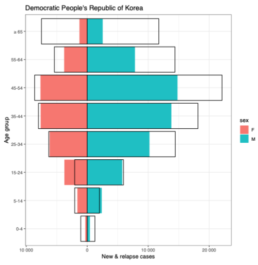	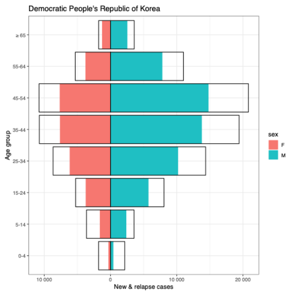

Figure S 7: *The countries where Method 2 did not result in acceptable estimates*

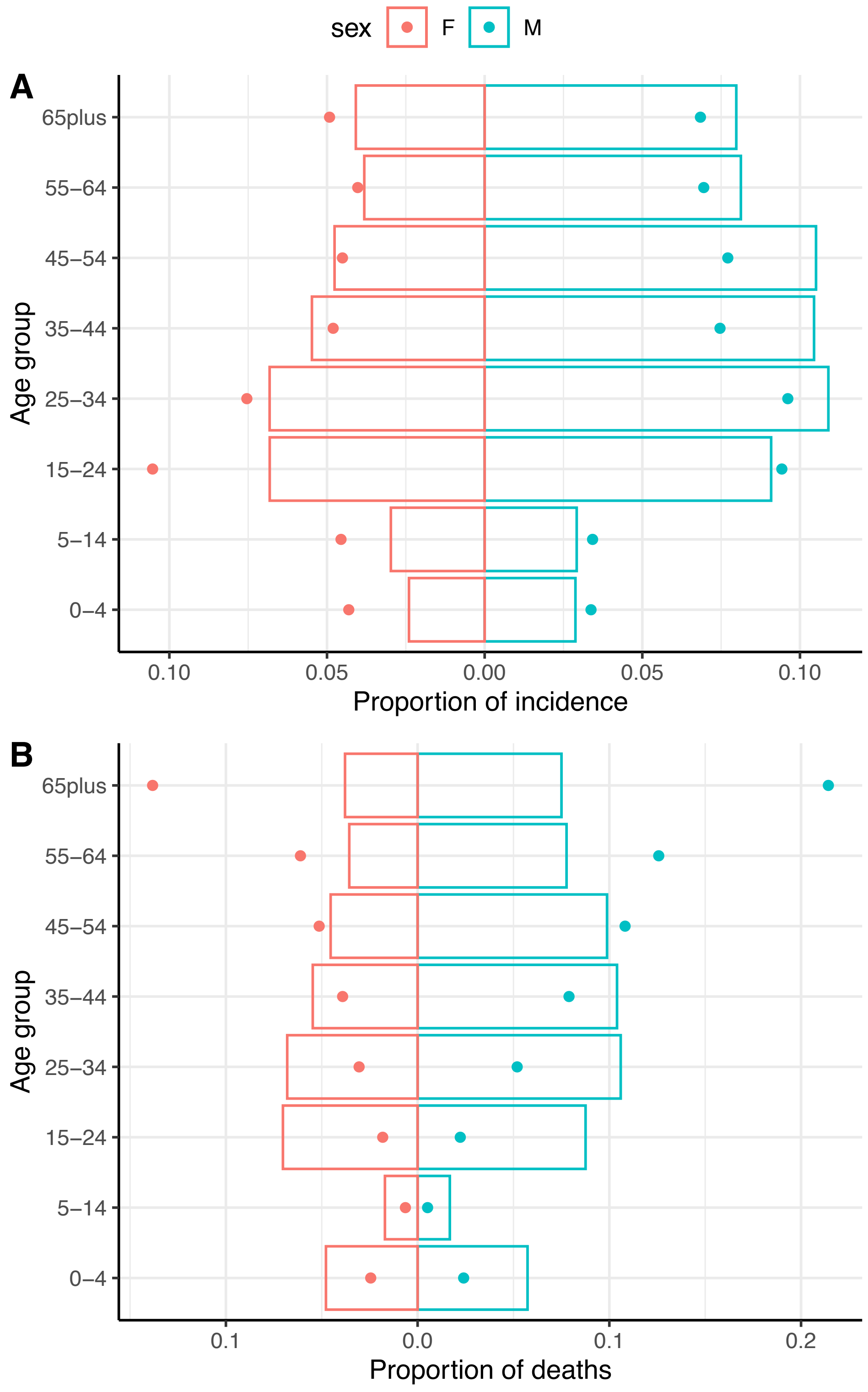

Figure S 8: *Comparison of IHME (dots, estimates for 2017) and WHO (bars, estimates for 2018) patterns of TB incidence (panel A) and deaths (panel B) by age and sex.*

The proportions of events in males for WHO vs IHME are: 63% vs 55% for incidence; 62% vs 63% for deaths. The proportions of events in children under 15 years for WHO vs IHME are: 11% vs 16% for incidence; 14% vs 6% for deaths.

Code and data for analysis: https://github.com/petedodd/sagediss
